# Effect of trabeculectomy on visual field progression in Japanese progressive normal-tension glaucoma with intraocular pressure < 15 mmHg

**DOI:** 10.1371/journal.pone.0184096

**Published:** 2017-08-29

**Authors:** Tomoko Naito, Miyuki Fujiwara, Takako Miki, Ryoichi Araki, Atsushi Fujiwara, Yusuke Shiode, Yuki Morizane, Mikio Nagayama, Fumio Shiraga

**Affiliations:** 1 Department of Ophthalmology, Okayama University Medical School Hospital, Okayama, Japan; 2 Nagayama Eye Clinic, Okayama, Japan; Massachusetts Eye & Ear Infirmary, Harvard Medical School, UNITED STATES

## Abstract

We examined the effectiveness of trabeculectomy in decreasing the slope of mean deviation (MD) in Japanese patients with progressive normal-tension glaucoma (NTG) at low intraocular pressure (IOP) levels. The charts of patients who had undergone initial trabeculectomy with adjunctive mitomycin C for progressive NTG with medically controlled IOP < 15 mmHg in 2010–2013 were retrospectively reviewed. Seventeen eyes of 13 NTG patients who had undergone at least 5 times of visual field (VF) examinations in both of preoperatively and postoperatively with postoperative follow-up of ≥ 2 years were enrolled. Preoperative and postoperative MD slopes were compared to evaluate the effectiveness of trabeculectomy in slowing progression of VF. Mean IOP (8.1 ± 2.9 mmHg) and number of IOP-lowering medications (0.8 ± 1.5) were significantly lower postoperatively than preoperatively (13.9 ± 0.9 mmHg; *P* < 0.001 and 3.0± 0.4; *P* < 0.0001). In total, 91.7% of eyes with single-digit IOP postoperatively showed improvement in MD slope, whereas only 20.0% of eyes with IOP ≥ 10 mmHg postoperatively showed the improvement. Three eyes (17.6%) showed a decrease in visual acuity (VA) of ≥ 0.1 unit; this group had a lower mean postoperative IOP (6.0 ± 1.0 vs. 8.6 ± 3.0 mmHg; *P* = 0.1717) and a higher mean IOP reduction rate (56.2 vs. 38.5%; *P* = 0.8296) than eyes with a VA decrease of < 0.1 unit or no change. Thus, in this analysis of Japanese NTG patients with medically controlled IOP < 15 mmHg, achieving an IOP < 10 mmHg with trabeculectomy was beneficial for reducing the VF progression rate in progressive NTG at low IOP levels. However, an IOP < 7 mmHg by surgery would be required careful attention to VA decline.

## Introduction

Glaucoma is currently the leading cause of blindness in Japan [1]. The slowing of visual field (VF) damage as a consequence of reduction of intraocular pressure (IOP) in primary open-angle glaucoma (POAG) has been established by randomized clinical trials [2–7]. However, in some cases, the VF damage continues to progress even if the IOP is brought to low double-digit figures by medication. In such cases, additional intervention may be needed to reach an IOP below the episcleral venous pressure. Trabeculectomy is an effective strategy for achieving an IOP reduction of 20%–40% even in normal-tension glaucoma (NTG) with VF progression at very low preoperative IOP levels [8–17]. Aoyama et al [8] reported that IOP was lowered from 15.2 mmHg to 9.4 mmHg by trabeculectomy in 40 eyes with NTG; 91.5% of the eyes with postoperative IOP < 10 mmHg had no VF progression over 15.4 years, whereas only 30.9% of those with postoperative IOP > 10 mmHg had no progression over 13.4 years. These authors recommended an IOP reduction of ≥ 20% from baseline or achievement of IOP < 10 mmHg as the postoperative IOP target [8]. Schultz et al [9,10] reported that trabeculectomy was an effective method for achieving single-digit IOP targets in progressive NTG with an IOP of < 15 mmHg.

Meanwhile, Japanese people have lower average IOP than Western people, and the average IOP of Japanese people is 14.5 mmHg (the normal range of the IOP is 10–20 mmHg) according to the results of the Tajimi study (a population-based prevalence survey of glaucoma by the Japan Glaucoma Society) [18,19]. NTG is the most frequent glaucoma (the prevalence rate is 3.6%) [18,19], and the IOP of Japanese patients with NTG is 11.5–11.6 mmHg on the lowest average and 16.6–16.7 mmHg on the highest average [20,21], which is lower than that in Western patients [22,23]. Furthermore, the frequency of shallow anterior chamber is high [24]. These are characteristics of Japanese people which is different from Western people and changed our perception of glaucoma. The IOP of the treatment target, which is the IOP level that does not deteriorate the optic nerve, can be considered to be different for each individual. However, there is currently no way to know the threshold level. The result of Collaborative Normal-Tension Glaucoma Study Group (CNTGS), which examined the relationship between VF progression and the IOP reduction therapy in NTG, showed that the rate of no progression of VF was 80% in the eyes with IOP reduction by 30% or more [22,23]. Almost half of the treatment group in this study had undergone surgery, and the average IOP after surgery was 10.6 mmHg. But it is unknown that safety and efficacy of lowering IOP to single digit in progressive NTG with below 15 mmHg under treatment.

Additionally, the presence of risk factors other than IOP factors is also suspected in the pathology of NTG. In the report of CNTGS [22,23], 20% of eyes in the treated group had VF progression despite adequate IOP reduction therapy, and 40% of eyes in the non-treated group had no progression in 5 years.

Several previous studies have reported that trabeculectomy slowed down progression of NTG [8–17], but in most of these studies, trabeculectomy was conducted in patients with relatively high IOP exceeding 15 mmHg. The unique feature of our study is that we performed trabeculectomy in patients who were traditionally not indicated for surgery; i.e. patients whose IOP was lowered to low double-digit figure (below 15 mmHg) by intensive medication, and examined the effectiveness of trabeculectomy in ameliorating glaucoma progression, and safety of achieving single-digit IOP.

The aims of this study were: to assess the efficacy of trabeculectomy with adjunctive mitomycin C (MMC) in decreasing the mean deviation (MD) slope in Japanese patients with progressive NTG at IOP < 15 mmHg; to identify a target IOP level in these eyes by comparing the preoperative and postoperative MD slopes; and to identify clinical factors contributing to non-improvement of the MD slope and deterioration in visual acuity (VA) after surgery.

## Patients and methods

### Study design and patients

This study was approved by the Ethical Committee of Okayama University (approval number: 1606–507). Informed consent was obtained from the subjects after a thorough explanation of the study objective and information collection was given in accordance with ethical principles based on the Helsinki Declaration. The study is registered with the UMIN Clinical Trial Registry (Trial Registration: UMIN000024929).

We retrospectively reviewed the consecutive clinical records of 249 eyes of 205 Japanese patients who had undergone initial trabeculectomy with adjunctive MMC for progressive open-angle glaucoma between 2010 and 2013 at Okayama University Hospital and had been followed postoperatively for more than 2 years. The type of glaucoma was diagnosed according to the guidelines of the Japan Glaucoma Society. [24]

The study inclusion criteria were as follows: a diagnosis of open angle glaucoma; a mean preoperative IOP < 15 mmHg under treatment; reliable VF results (fixation loss < 20%, false positive < 15%, and false negative < 33%); and VF testing on at least 5 occasions in both of preoperative and postoperative surgery. The exclusion criteria were as follows: ocular disease that might affect visual function other than glaucoma; prior eye surgery other than for cataract; and any complications that might influence the VF defect.

### Surgical procedure

Trabeculectomy was performed in a standardized manner with a fornix-based flap of the conjunctiva and Tenon’s capsule by one glaucoma surgeon (T.N.). A half-thickness 4 mm × 4 mm scleral flap was dissected to the clear cornea. A fluid-retaining sponge soaked with MMC (0.4 mg/mL) was applied to the superior sclera for 5 minutes, followed by washing with 250 mL of saline solution. After excision of the trabeculum, a peripheral iridectomy was performed. The scleral flap and conjunctiva were sutured firmly with 10–0 nylon, and Seidel testing was performed at the end of the procedure. Cataract surgery was performed in combination with trabeculectomy as appropriate. Administration of topical ocular hypotensive drugs after surgery was allowed if necessary.

### Ophthalmic examinations

Ophthalmic examinations were conducted by the surgeon who performed the trabeculectomies and three ophthalmologists. Patients was routinely followed approximately four times a year, with more frequent visits during the early postoperative period. Due to the presence of diurnal fluctuation in IOP, care was taken to standardize the time of measurement. Slit-lamp examination and IOP measurement were performed at each visit. Patients were instructed to visit the clinic at around the same time. The time of IOP measurement was recorded. VF was evaluated every 6 months pre and postoperatively. The best-corrected visual acuity (BCVA) was measured every 6 months.

The IOP measurement was performed using the Goldmann applanation tonometer. The IOP reduction rate (%) was defined as preoperative IOP minus postoperative IOP divided by preoperative IOP, which was multiplied by 100. The VF examinations were performed using the Humphrey VF Analyzer (Carl Zeiss Meditec Inc., Dublin, CA, USA) with the standard 30–2 or 10–2 test pattern, Swedish Interactive Threshold Algorithm (SITA) standard strategy. Progression of VF was evaluated using trend-based analysis, which generates the rate of progression based on changes in MD over time (HfaFiles ver.5, Beeline Co., Ltd, Tokyo, Japan). Preoperative and postoperative MD slopes were compared to evaluate the effectiveness of trabeculectomy in decreasing the MD slope. The subject eyes were also classified into two groups according to the presence or absence of improvement in the MD slope at the final examination after surgery, and differences in the clinical characteristics of the two groups were evaluated. The preoperative MD slope value that could predict 50% improvement in the slope by trabeculectomy was identified by receiver-operating characteristic (ROC) analysis and the decision-tree approach respectively.

The BCVA measured using a standard Japanese decimal VA chart was recorded, and the mean BCVA was calculated using the logarithm of the minimal angle of resolution (logMAR) scale. Permanent impairment of VA was defined as a deterioration in VA of at least 0.1 unit of logMAR at a minimum of 2 consecutive examinations until the final observation.

The central corneal thickness (CCT) was measured using the specular microscope EM-3000^®^ (Tomey Corp., Nagoya, Japan), and the axial length was measured using the non-contact optical biometer OA-2000^®^ (Tomey Corp., Nagoya, Japan).

### Statistical analysis

The statistical analysis was performed using JMP software version 8.0 (SAS Institute Inc., Cary, NC, USA). An unpaired *t*-test was applied for continuous variables and comparison between groups. The ROC analysis was performed using AccuROC 2.5 software (Accumetric Corp., Montreal, QC, Canada). The ROC for the preoperative MD slope values was plotted to determine the optimum cut-off point, and the area under the ROC curve was calculated to determine the power of discrimination between eyes with and without 50% improvement in MD slope after surgery. Decision trees were constructed by the decision-tree analysis, and the cut-off value for the preoperative MD slope was estimated by separating the cases with 50% improvement in MD slope after surgery from those without 50% improvement (JMP Ver.12, SAS Institute Inc., Cary, NC, USA). The results were expressed as the mean ± standard deviation. The significance level was set at *P* < 0.05.

## Results

### Preoperative data

Of the initial 249 consecutive eyes reviewed, 217 eyes were excluded because they had ocular pressure higher than 15 mmHg. Another 11 cases were excluded because they did not have at least 5 reliable visual field examination results before and after trabeculectomy. Only 21 eyes (17 cases of NTG and 4 cases of exfoliative glaucoma (XGF)) satisfied all the inclusion criteria. The 4 cases of XGF were further excluded because NTG and XGF are distinct pathologies and cannot be analyzed together. Eventually, 17 cases of NTG were analyzed. The average preoperative follow-up period of 5.0 ± 1.3 years available for analysis.

The baseline demographic and clinical characteristics for the 17 eyes of 13 patients were presented in Table 1. The mean preoperative IOP was 13.9 ± 0.9 mmHg and the mean number of IOP-lowering eye drops used preoperatively was 3.0 ± 0.4. Thirteen of the 17 eyes were examined using the 30–2 VF test, 6 using the 10–2 VF test, and 2 using both tests. The mean preoperative MD was -18.86 ± 4.16 dB with the 30–2 VF test (n = 13) and -28.81 ± 3.20 dB with the 10–2 VF test (n = 6); the respective mean preoperative MD slopes were -0.91 ± 0.57 dB/year and -0.97 ± 0.70 dB/year respectively. Four eyes had a history of cataract surgery and 12 eyes underwent cataract surgery performed in combination with trabeculectomy.

**Table 1:** Baseline demographic and clinical characteristics.

Subjects		17 eyes of 13 NTG patients [range]
**Age (years)**		69.5 ± 7.6 [51 to 83]
**Sex**	**Female** **Male**	9 eyes of 7 patients 8 eyes of 6 patients
**Preoperative IOP (mmHg)**		13.9 ± 0.9 [12 to 15]
**Number of IOP-lowering eye drops**		3.0 ± 0.4 [2 to 4]
**Preoperative MD** **(dB)**	**30–2 VF test (n = 13)** **10–2 VF test (n = 6)**	-18.86 ± 4.16 [-27.05 to -12.49] -28.81 ± 3.20 [-32.99 to -25.29]
**Preoperative MD slope** **(dB/year)**	**30–2 VF test (n = 13)** **10–2 VF test (n = 6)**	-0.91 ± 0.57 [-2.18 to -0.05] -0.97 ± 0.70 [-2.18 to -0.08]
**CCT (μm)**		505.4 ± 51.11 [412 to 575]
**Axial length (mm)**		24.07 ± 1.53 [22.20 to 25.27]
**History of cataract surgery: Yes**		4 eyes of 17 patients
**Concurrent cataract surgery: Yes**		12 eyes of 17 patients

The data were shown as the mean ± standard deviation. Abbreviations: NTG, normal-tension glaucoma; IOP, intraocular pressure; MD, mean deviation; CCT, central corneal thickness

### Postoperative data

Changes in IOP, medication, MD, and VA after surgery were shown in Table 2. The mean duration of follow-up was 5.0 ± 1.3 years after surgery. At the time of the final visit, the mean postoperative IOP (8.1± 2.9mmHg) was significantly lower than the mean preoperative IOP (13.9 ± 0.9 mmHg; *P* < 0.001). This represented a mean reduction in IOP of 5.8 mmHg (41.7%) from the preoperative IOP level. Twelve (70.6%) of 17 eyes achieved an IOP < 10 mmHg, and 14 (82.4%) of 17 eyes achieved an IOP reduction of > 20% from the preoperative IOP level. The mean number of IOP-lowering eye drops used was also significantly lowered from 3.0 ± 0.4 to 0.8 ± 1.5 (*p* < 0.0001). The rate of IOP decrease was not significantly different between the group that underwent trabeculectomy combined with cataract surgery (n = 12) and the group that underwent trabeculectomy alone (n = 5) (40.02 ± 21.1% vs 43.38 ± 21.2%, *p* = 0.8296).

**Table 2:** IOP, medication, MD, and VA before and after surgery (n = 17).

Variable	Before surgery ^#1^	After surgery ^#2^	*P*-value ^#3^
**Follow-up duration (years)**	4.3 ± 1.7	5.0 ± 1.3	-
**IOP (mmHg)**	13.9 ± 0.9	8.1 ± 2.9	< 0.001*
**Rate of IOP reduction (%)**	-	41.7 ± 20.5	-
**Number of IOP-lowering eye drops (n)**	3.0 ± 0.4	0.8 ± 1.5	< 0.0001*
**Number of VF examinations (times)**			
30–2 VF test (n = 13)	7.6 ± 3.5	6.9 ± 2.2	-
10–2 VF test (n = 6)	7.8 ± 1.7	6.7 ± 1.6	-
**MD value (dB)**			
30–2 VF test (n = 13)	-18.86 ± 4.16	-20.11 ± 4.25	0.2901
10–2 VF test (n = 6)	-28.81 ± 3.20	-28.43 ± 2.36	0.2242
**MD slope (dB/year)**			
30–2 VF test (n = 13)	-0.91 ± 0.57	-0.32 ± 0.57	0.0334*
10–2 VF test (n = 6)	-0.97 ± 0.70	-0.09 ± 0.37	0.0709
**logMAR VA**	0.23 ± 0.45	0.17 ± 0.27	0.4845
**Decimal VA**	0.78 ± 0.40	0.79 ± 0.40	0.8974

The data were shown as the mean ± standard deviation.

*Statistically significant values.

^#1^Mean values of subject eyes at final measurement before surgery;

^#2^mean values of subject eyes at final follow-up after surgery;

^#3^paired *t*-test. Abbreviations: IOP, intraocular pressure; NTG, normal-tension glaucoma; VF, visual field; MD, mean deviation; logMAR, logarithm of minimum angle of resolution

The mean number of VF examinations was 7.6 ± 3.5 with the 30–2 VF test and 7.8 ± 1.7 with the 10–2 VF test preoperatively, and 6.9 ± 2.2 with the 30–2 VF test and 6.7 ± 1.6 with the 10–2 VF test postoperatively. The mean postoperative MD slope using the 30–2 VF test (-0.32 ± 0.57 dB/year) was significantly improved when compared with the mean preoperative MD slope (-0.91 ± 0.57 dB/year; *P* = 0.0334), while there no statistically significant difference, the postoperative MD slope using the 10–2 VF test (-0.09 ± 0.37 dB/year) improved when compared with the preoperative MD slope (-0.97 ± 0.70 dB/year; *P* = 0.070). There was no significant difference in mean VA before and after surgery.

Postoperative complications were shown in Table 3. There were no cases of surgical failure indicated by need for reoperation for glaucoma or loss of light perception. Overall, 12 (70.6%) of 17 eyes experienced postoperative complications as follows: transient hypotony (IOP < 5 mmHg was observed once or more at follow-up visits after surgery) in 9eyes (52.9%), a VA decline by ≥ 0.1 unit in 3eyes (17.6%), hypotony maculopathy in 3 eyes (17.6%), hyphema in 4 eyes (23.5%), choroidal detachment in 4 eyes (23.5%), shallow anterior chamber in 2 eyes (11.8%), and leakage of aqueous humor in 1 eye (5.9%).

**Table 3:** Postoperative complications.

	17 eyes of 13 patients
Total postoperative complications	12 eyes (70.6%)
Transient hypotony ^#^	9 eyes (52.9%)
VA decline by ≥ 0.1 unit	3 eyes (17.6%)
Hypotony maculopathy	3 eyes (17.6%)
Hyphema	4 eyes (23.5%)
Choroidal detachment	4 eyes (23.5%)
Shallow anterior chamber	2 eyes (11.8%)
Aqueous humor leakage	1 eye (5.9%)

^#^IOP < 5 mmHg was observed once or more at follow-up visits after surgery.

**Fig 1:**
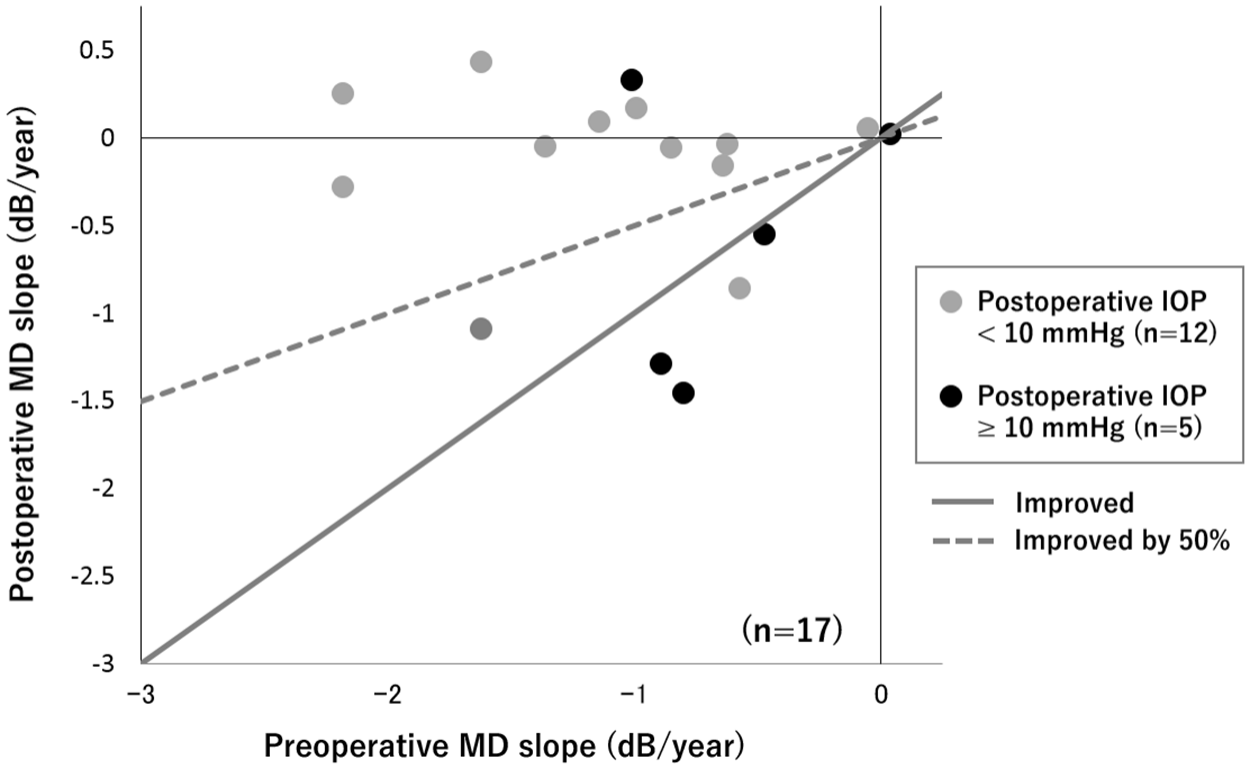
Scatterplot of preoperative MD slope versus postoperative MD slope. The gray circles indicated a postoperative IOP < 10 mmHg. The black circles indicated a postoperative IOP ≥ 10 mmHg. The gray line indicated no difference between the preoperative and postoperative MD slopes. The dotted line indicated a 50% improvement in the postoperative MD slope. The preoperative MD slope value was the measurement recorded at the final follow-up before surgery. The postoperative MD slope and postoperative IOP values were those recorded at the final follow-up after surgery. Abbreviations: MD, mean deviation, IOP, intraocular pressure.

A scatterplot of the preoperative MD slope versus the postoperative MD slope for all eyes was shown in Fig 1. The proportion of eyes with improvement in MD slope was 70.6% (12/17 eyes). When the eyes were divided according to whether they had a postoperative IOP < 10 mmHg (single-digit IOP group) or ≥ 10 mmHg (double-digit IOP group), 11 (91.7%) of the 12 eyes in the single-digit IOP group showed improvement in MD slope whereas only 1 (20.0%) of the 5 eyes in the double-digit IOP group showed the improvement. Using the ROC analysis, we identified the optimal cut-off value on the preoperative MD slope that could predict 50% improvement in MD slope after trabeculectomy to be -0.91 dB/year (sensitivity: 0.6875, specificity: 1.0); when using the decision-tree approach, the cut-off value was -0.89 dB/year.

A comparison of clinical characteristics according to the presence or absence of improvement in the MD slope is shown in Table 4. Compared with the group showing improvement in MD slope, the group showing no improvement in MD slope had a significantly higher mean postoperative IOP (6.8 ± 2.0 mmHg vs. 11.2 ± 2.4 mmHg; *P* = 0.0016) and a significantly lower mean rate of reduction in IOP (50.0 ± 17.3% vs. 21.7 ± 12.5%; *P* = 0.0050). The group showing improvement in MD slope used a significantly lower mean number of IOP-lowering eye drops after surgery than the group showing no improvement in MD slope (0.3 ± 1.2 vs. 2.0 ± 1.6; *P* = 0.0275). Although hyphema was observed in 1 eye in the group showing no improvement in MD slope, no complications were observed in the remaining 4 eyes. No significant difference in patient age, CCT<, or axial length was seen between the two groups.

**Table 4:** Comparison between groups with or without improvement in MD slope.

Variable		MD slope		*P*-value ^#3^
		Not improved ^#1^ (n = 5)	Improved ^#2^ (n = 12)	
**IOP (mmHg)**	Before surgery	14.2 ± 0.8	13.8 ± 0.9	0.4616
	After surgery	11.2 ± 2.4	6.8 ± 2.0	0.0016*
**Rate of IOP reduction (%)**		21.7± 12.5	50.0± 17.3	0.0050*
**IOP-lowering** **eye drops (n)**	Before surgery	2.8 ± 0.4	3.1 ± 0.3	0.1365
	After surgery	2.0 ± 1.6	0.3 ± 1.2	0.0275*
**Postoperative complications: Yes (n**				
Transient hypotony ^#4^		0	9	-
VA decline by ≥ 0.1 unit		0	3	-
Hyphema		1	3	-
Choroidal detachment		0	4	-
Shallow anterior chamber		0	2	-
Aqueous humor leakage		0	1	-
**Age (years)**		70.1 ± 7.0	69.4 ± 7.7	0.9656
**CCT (μm)**		479.0 ± 59.8	518.6 ± 43.5	0.1511
**Axial length (mm)**		23.2 ± 1.3	24.4 ± 1.5	0.1647

The data were shown as the mean ± standard deviation.

*Statistically significant values. The mean values in each group at the final measurement before surgery were indicated by “before surgery” and those at the final follow-up after surgery are indicated by “after surgery”.

^#1^The MD slope at final follow-up after surgery was decreased or unchanged when compared with that at the final measurement before surgery.

^#2^The MD slope at final follow-up after surgery was improved when compared with the MD slope at the final measurement before surgery.

^#3^Unpaired *t-*test.

^#4^IOP < 5 mmHg was observed once or more at follow-up visits after surgery. Abbreviations: NTG, normal-tension glaucoma; IOP, intraocular pressure; CCT, central corneal thickness

A comparison of clinical characteristics according to whether VA declined by ≥ 0.1 unit (VA decline group) or < 0.1 unit (no change group) is shown in Table 5. Mean postoperative IOP was lower in the VA decline group than in the no change group (6.0 ± 1.0 mmHg vs. 8.6 ± 3.0 mmHg; *P* = 0.1717), but there was no statistically significant difference. There was no significant difference in mean preoperative VA (0.83 ± 0.15 vs. 0.77 ± 0.44; *P* = 0.8226) or mean preoperative IOP (13.7 ± 0.6 mmHg vs. 14.0 ± 1.0 mmHg; *P* = 0.5770) between the two groups, as well as in the mean number of IOP-lowering eye drops used, age, CCT, or axial length. The complications observed in the VA decline group comprised transient hypotony (3 eyes) and hypotony maculopathy (3 eyes).

**Table 5:** Comparison between the groups with or without a decline in VA.

Variables		VA		*P*-value ^#4^
		Decline (n = 3) ^#1^	No change (n = 14) ^#2^	
**Decimal VA**	Before surgery	0.83 ± 0.15	0.77 ± 0.44	0.8226
	After surgery	0.57 ± 0.21	0.84 ± 0.42	0.2978
**IOP (mmHg)**	Before surgery	13.7 ± 0.6	14.0 ± 1.0	0.5770
	After surgery	6.0 ± 1.0	8.6 ± 3.0	0.1717
**Rate of IOP reduction (%)**		56.2 ± 5.8	38.5 ± 21.3	0.1825
**IOP-lowering** **eye drops (n)**	Before surgery	3.0 ± 0.0	3.0 ± 0.4	1.0000
	After surgery	0.0 ± 0.0	1.0 ± 1.6	0.2989
**Age (years)**		66.7 ± 12.7	70.1 ± 6.4	0.4996
**CCT (μm)**		549.0 ± 38.3	494.5 ± 49.1	0.0995
**Axial length (mm)**		24.5 ± 2.4	24.0 ± 1.4	0.6008
**Postoperative complications:** **Yes (n)** ^#3^		3	9	-

The data were presented as the mean ± standard deviation. The mean values of each group at the final measurement before surgery were indicated by “before surgery” and those at the final follow-up after surgery were indicated by “after surgery”.

^#1^The VA at final follow-up after surgery was lower by ≥ 0.1 unit when compared with the VA at the final measurement before surgery.

^#2^The VA at the final follow-up after surgery was lower by <0.1 unit or unchanged when compared with the VA at the final measurement before surgery.

^#3^Transient hypotony of < 5 mmHg, hyphema, choroidal detachment, shallow anterior chamber, aqueous humor leakage.

^#4^Unpaired *t-*test. Abbreviations: VA, visual acuity; NTG, normal-tension glaucoma; IOP, intraocular pressure; CCT, central corneal thickness

## Discussion

In the present study, the mean IOP was significantly decreased from 13.9 mmHg to 8.1 mmHg by trabeculectomy with MMC, and the IOP reduction rate was 41.7%. These results appear to be comparable with previous reports. [8–17] Iverson et al [10] demonstrated a decrease in IOP from 13.1 mmHg to 8.5 mmHg and an increase in the MD slope from -1.05 dB/year to -0.25 dB/year over a follow-up period of approximately 5.9 years in 14 eyes with progressive NTG that underwent trabeculectomy with MMC. Koseki et al [11] reported that IOP was lowered from 16 mmHg to 9.2 mmHg and the MD slope was increased from -1.48 dB/year to +0.13 dB/year at 2 years by trabeculectomy with antimetabolites in 21 eyes with progressive NTG. Daugeliene et al [13] reported that trabeculectomy with MMC decreased the MD slope from -0.97 dB/year to -0.32 dB/year, with an IOP reduction from 14.7 mmHg to 8.7 mmHg. Cataract surgery alone has been reported to decrease IOP [25]. In the present study, the IOP-lowering effect of trabeculectomy was not different between the group that underwent simultaneous cataract surgery and the group that did not. This result suggests that simultaneous cataract surgery has no remarkable effect on the postoperative outcome of trabeculectomy.

Moreover, in the scatterplot for preoperative MD slope versus postoperative MD slope, all the eyes with a relatively lower preoperative MD slope (lower than -1.0 dB/year) showed improvement in the postoperative MD slope. The ROC analysis and decision-tree method revealed that a preoperative MD slope of -0.9 dB/year was a predictor of 50% improvement in MD slope after surgery. Thus, in eyes with NTG that are progressing at low IOP levels, rapid progressive eyes with a MD slope of < -0.9 dB/year could achieve a 50% improvement in MD slope if treated by trabeculectomy.

Meanwhile, 5 eyes were identified as having a worse or unchanged MD slope after surgery. Glaucomatous VF progression could still continue in some eyes with NTG even at an IOP level < 10 mmHg, suggesting that the VF changes might be partly related to factors other than IOP in such cases. Daugeliene et al [13] constructed scatterplots to visualize the relationship between the preoperative and postoperative MD slopes in 32 trabeculectomized eyes with NTG (9 eyes with a mean IOP ≥ 15 mmHg, 23 eyes with a mean IOP < 15 mmHg). The plots indicated that the postoperative MD slope was improved without exception in eyes with a preoperative mean IOP ≥ 15 mmHg; however, there were cases of a worse or unchanged MD slope after surgery in eyes with a preoperative mean IOP < 15 mmHg. [13]

As with all surgical procedures, the benefits of aggressive IOP lowering can be interpreted in the context of the associated adverse events. We observed a VA decline by ≥ 0.1 unit in 3eyes (17.6%) in this study (a loss of 0.1 unit in 2 eyes and a loss of 0.2 units in 1 eye). There was no statistically significant difference in mean postoperative IOP between the VA decline group and the no change group (6.0 ± 1.0 mmHg vs. 8.6 ± 3.0 mmHg; *P* = 0.1717), however, mean postoperative IOP was lower and mean reduction rate of IOP was higher in the VA decline group than in the no change group. Because hypotony maculopathy was observed in 3 eyes with VA decline, we cannot exclude the possibility that the decline in vision occurred as a consequence of ocular rigidity. This may suggest that even if single-digit IOP has the beneficial effect of decreasing glaucomatous VF progression, an IOP < 7.0 mmHg could lead to an increased incidence of VA decline that decreases the quality of vision. Thus, maintaining a postoperative IOP of ≥ 7 mmHg is preferable for avoiding a decline in VA. Further studies are needed to elucidate the potential IOP threshold to avoid a marked VA decline after trabeculectomy.

Our study has some limitations. First, our study had a retrospective design, and a small number of only 17 cases of NTG were included in analysis. Second, there was a potential for selection bias given that surgery was not performed in a randomized masked manner. Third, some patients were followed by 30–2 VF test while others by 10–2 test. The reason is that a large proportion of the patients had advanced glaucoma, and patients with threatened central visual function were followed by 10–2 test. However, regardless of 30–2 or 10–2, glaucoma progression was ameliorated as IOP declined. Fourth, this study did not include data on disc hemorrhage (DH) or IOP fluctuations. Komori et al [26] reported that DH and IOP fluctuation were associated with VF progression in NTG. It has also been reported that eyes with glaucoma that had undergone trabeculectomy showed less diurnal IOP fluctuation than eyes with medically treated glaucoma [27,28] and that trabeculectomy reduced the degree of posture-induced IOP changes in OAG. [29–33] Sawada et al [31] reported that eyes with successfully trabeculectomized OAG showed smaller posture-induced IOP changes than eyes with non-operated, medically treated OAG, despite the mean IOP in both groups being the same (9.7 mmHg). Given that trabeculectomy creates a new aqueous pathway via the filtering bleb that is independent of the episcleral veins, it seems reasonable that the surgery could cause suppression of posture-induced IOP alterations.

In conclusion, we performed trabeculectomy in patients who were traditionally not indicated for surgery; i.e. patients whose IOP was lowered to low double-digit figure (below 15 mmHg) by intensive medication, and confirmed that glaucoma progression was ameliorated. our results showed that achieving single-digit IOP by trabeculectomy has a beneficial effect of delaying progression of the VF defect in Japanese patients with progressive NTG. There was a possibility that the rate of progression would be halved by trabeculectomy in eyes with a preoperative MD slope of < -0.9 dB/year. However, considering that postoperative complications may arise and cause some visual disturbance, the decision regarding filtering surgery should be made on an individual basis, with understanding of patient-specific characteristics such as age, life expectancy, comorbidities, and the baseline level of damage.

## References

[1] Yamada M, Hiratsuka Y, Roberts CB, Pezzullo ML, Yates K, Takano S, et al Prevalence of visual impairment in the adult Japanese population by cause and severity and future projections. Ophthalmic Epidemiol. 2010;17(1):50–57. doi: 10.3109/09286580903450346 20100100 10.3109/09286580903450346

[2] Gordon MO, Beiser JA, Brandt JD, Heuer DK, Higginbotham EJ, Johnson CA, et al The Ocular Hypertension Treatment Study: baseline factors that predict the onset of primary open-angle glaucoma. Arch Ophthalmol. 2002;120(6):714–720. 12049575 10.1001/archopht.120.6.714

[3] Leske MC, Heijl A, Hyman L, Bengtsson B, Dong L, Yang Z; EMGT Group. Predictors of long-term progression in the early manifest glaucoma trial. Ophthalmology. 2007;114(11):1965–1972. doi: 10.1016/j.ophtha.2007.03.016 17628686 10.1016/j.ophtha.2007.03.016

[4] Musch DC, Gillespie BW, Lichter PR, Niziol LM, Janz NK; CIGTS Study Investigators. Visual field progression in the Collaborative Initial Glaucoma Treatment Study the impact of treatment and other baseline factors. Ophthalmology. 2009;116(2):200–207. doi: 10.1016/j.ophtha.2008.08.051 19019444 10.1016/j.ophtha.2008.08.051 PMC3316491

[5] Drance S, Anderson DR, Schulzer M; Collaborative Normal-Tension Glaucoma Study Group. Risk factors for progression of visual field abnormalities in normal-tension glaucoma. Am J Ophthalmol. 2001;131(6):699–708. 11384564 10.1016/s0002-9394(01)00964-3

[6] Miglior S, Torri V, Zeyen T, Pfeiffer N, Vaz JC, Adamsons I; EGPS Group. Intercurrent factors associated with the development of open-angle glaucoma in the European glaucoma prevention study. Am J Ophthalmol. 2007;144(2):266–275. doi: 10.1016/j.ajo.2007.04.040 17543874 10.1016/j.ajo.2007.04.040

[7] [No authors listed]. The Advanced Glaucoma Intervention Study (AGIS): 7. The relationship between control of intraocular pressure and visual field deterioration. The AGIS Investigators. Am J Ophthalmol. 2000;130(4):429–440. 11024415 10.1016/s0002-9394(00)00538-9

[8] Aoyama A, Ishida K, Sawada A, Yamamoto T. Target intraocular pressure for stability of visual field loss progression in normal-tension glaucoma. Jpn J Ophthalmol. 2010;54(2):117–123. doi: 10.1007/s10384-009-0779-z 20401559 10.1007/s10384-009-0779-z

[9] Schultz SK, Iverson SM, Shi W, Greenfield DS. Safety and efficacy of achieving single-digit intraocular pressure targets with filtration surgery in eyes with progressive normal-tension glaucoma. J Glaucoma. 2016;25(2):217–222. doi: 10.1097/IJG.0000000000000145 25264998 10.1097/IJG.0000000000000145 PMC4375069

[10] Iverson SM, Schultz SK, Shi W, Feuer WJ, Greenfield DS. Effectiveness of single-digit IOP targets on decreasing global and localized visual field progression after filtration surgery in eyes with progressive normal-tension glaucoma. J Glaucoma. 2016;25(5):408–414. doi: 10.1097/IJG.0000000000000240 25719235 10.1097/IJG.0000000000000240

[11] Koseki N, Araie M, Shirato S, Yamamoto S. Effect of trabeculectomy on visual field performance in central 30 degrees field in progressive normal-tension glaucoma. Ophthalmology. 1997;104(2):197–201. 9052622 10.1016/s0161-6420(97)30334-0

[12] Shigeeda T, Tomidokoro A, Araie M, Koseki N, Yamamoto S. Long-term follow-up of visual field progression after trabeculectomy in progressive normal-tension glaucoma. Ophthalmology. 2002;109(4):766–770. 11927438 10.1016/s0161-6420(01)01009-0

[13] Daugeliene L, Yamamoto T, Kitazawa Y. Effect of trabeculectomy on visual field in progressive normal-tension glaucoma. Jpn J Ophthalmol. 1998;42(4):286–292. 9749869 10.1016/s0021-5155(98)00013-6

[14] Hagiwara Y, Yamamoto T, Kitazawa Y. The effect of mitomycin C trabeculectomy on the progression of visual field defect in normal-tension glaucoma. Graefes Arch Clin Exp Ophthalmol. 2000;238(3):232–236. 10796038 10.1007/s004170050349

[15] Jongsareejit B, Tomidokoro A, Mimura T, Tomita G, Shirato S, Araie M. Efficacy and complications after trabeculectomy with mitomycin C in normal-tension glaucoma. Jpn J Ophthalmol. 2005;49(3):223–227. doi: 10.1007/s10384-004-0181-9 15944828 10.1007/s10384-004-0181-9

[16] Membrey WL, Bunce C, Poinoosawmy DP, Fitzke FW, Hitchings RA. Glaucoma surgery with or without adjunctive antiproliferatives in normal tension glaucoma: 2 Visual field progression. Br J Ophthalmol. 2001;85(6):696–701. doi: 10.1136/bjo.85.6.696 11371491 10.1136/bjo.85.6.696 PMC1724011

[17] Bhandari A, Crabb DP, Poinoosawmy D, Fitzke FW, Hitchings RA, Noureddin BN. Effect of surgery on visual field progression in normal-tension glaucoma. Ophthalmology. 1997;104(7):1131–1137. 9224466 10.1016/s0161-6420(97)30172-9

[18] Iwase A, Suzuki Y, Araie M, Yamamoto T, Abe H, Shirato S, et al The prevalence of primary open-angle glaucoma in Japanese: the Tajimi Study. Ophthalmology. 2004;111(9):1641–1648. doi: 10.1016/j.ophtha.2004.03.029 15350316 10.1016/j.ophtha.2004.03.029

[19] Yamamoto T, Iwase A, Araie M, Suzuki Y, Abe H, Shirato S, et al The Tajimi Study report 2: prevalence of primary angle closure and secondary glaucoma in a Japanese population. Ophthalmology. 2005;112(10):1661–1669. doi: 10.1016/j.ophtha.2005.05.012 16111758 10.1016/j.ophtha.2005.05.012

[20] Ishii Reiko, Yamagami Junkichi, Araie Makoto. Clinical implication of diurnal intraocular pressure curve in low tension glaucoma. Sinsho Ganka [Japanese Journal of clinical ophthalmology], 44(9);1445–1448,1990.

[21] Kitazawa Yoshiaki. Pathology and management of open-angle glaucoma. Nippon Ganka Gakkai Zasshi [Journal of Japanese Ophthalmological Society], 105(12): 828–842, 2001. 11802456

[22] Collaborative Normal-Tension Glaucoma Study Group. Comparison of glaucomatous progression between untreated patients with normal-tension glaucoma and patients with therapeutically reduced intraocular pressures. Collaborative Normal-Tension Glaucoma Study Group. Am J Ophthalmol. 1998;126(4):487–497. 9780093 10.1016/s0002-9394(98)00223-2

[23] Collaborative Normal-Tension Glaucoma Study Group. The effectiveness of intraocular pressure reduction in the treatment of normal-tension glaucoma. Collaborative Normal-Tension Glaucoma Study Group. Am J Ophthalmol. 1998;126(4):498–505. 9780094 10.1016/s0002-9394(98)00272-4

[24] Japan Glaucoma Society. Guidelines for Glaucoma. Tokyo, Japan: Japan Glaucoma Society; 2002.

[25] Armstrong JJ, Wasiuta T, Kiatos E, Malvankar-Mehta M, Hutnik CM. The Effects of Phacoemulsification on Intraocular Pressure and Topical Medication Use in Patients With Glaucoma: A Systematic Review and Meta-analysis of 3-Year Data. J Glaucoma. 2017 doi: 10.1097/IJG.0000000000000643 [Epub ahead of print] 28333892 10.1097/IJG.0000000000000643

[26] Komori S, Ishida K, Yamamoto T. Results of long-term monitoring of normal-tension glaucoma patients receiving medical therapy: results of an 18-year follow-up. Graefes Arch Clin Exp Ophthalmol. 2014;252(12):1963–1970. doi: 10.1007/s00417-014-2767-3 25128961 10.1007/s00417-014-2767-3

[27] Medeiros FA, Pinheiro A, Moura FC, Leal BC, Susanna R Jr. Intraocular pressure fluctuations in medical versus surgically treated glaucomatous patients. J Ocul Pharmacol Ther. 2002;18(6):489–498. doi: 10.1089/108076802321021036 12537675 10.1089/108076802321021036

[28] Konstas AG, Topouzis F, Leliopoulou O, Pappas T, Georgiadis N, Jenkins JN, et al 24-hour intraocular pressure control with maximum medical therapy compared with surgery in patients with advanced open-angle glaucoma. Ophthalmology. 2006;113(5):761–5.e1. doi: 10.1016/j.ophtha.2006.01.029 16650670 10.1016/j.ophtha.2006.01.029

[29] Sawada A, Yamamoto T. Effects of trabeculectomy on posture-induced intraocular pressure changes over time. Graefes Arch Clin Exp Ophthalmol. 2012;250(9):1361–1366. doi: 10.1007/s00417-012-1942-7 22323246 10.1007/s00417-012-1942-7

[30] Kiuchi T, Motoyama Y, Oshika T. Relationship of progression of visual field damage to postural changes in intraocular pressure in patients with normal-tension glaucoma. Ophthalmology. 2006;113(12):2150–2155. doi: 10.1016/j.ophtha.2006.06.014 16996611 10.1016/j.ophtha.2006.06.014

[31] Sawada A, Yamamoto T. Comparison of posture-induced intraocular pressure changes in medically treated and surgically treated eyes with open-angle glaucoma. Invest Ophthalmol Vis Sci. 2014;55(1):446–450. doi: 10.1167/iovs.13-13030 24398092 10.1167/iovs.13-13030

[32] Weizer JS, Goyal A, Ple-Plakon P, Trzcinka A, Strong BD, Bruno CA, et al Bleb morphology characteristics and effect on positional intraocular pressure variation. Ophthalmic Surg Lasers Imaging. 2010;41(5):532–537. doi: 10.3928/15428877-20100726-06 20795573 10.3928/15428877-20100726-06

[33] Hirooka K, Takenaka H, Baba T, Takagishi M, Mizote M, Shiraga F. Effect of trabeculectomy on intraocular pressure fluctuation with postural change in eyes with open-angle glaucoma. J Glaucoma. 2009;18(9):689–691. doi: 10.1097/IJG.0b013e31819c49f4 20010249 10.1097/IJG.0b013e31819c49f4

